# Highly sensitive and non-disruptive detection of residual undifferentiated cells by measuring miRNAs in culture supernatant

**DOI:** 10.1038/s41598-022-14273-z

**Published:** 2022-06-20

**Authors:** Kanako Masumoto, Yuki Aihara, Mao Miyagawa Kuroishi, Natsuki Maeda, Yumiko Sakai, Yuma Oka, Yusuke Takahashi, Kenta Oda, Masatoshi Yanagida

**Affiliations:** Sysmex Corporation, Central Research Laboratories, 4-4-4 Takatsukadai, Nishi-ku, Kobe, 651-2271 Japan

**Keywords:** Stem cells, Pluripotent stem cells, Biotechnology, Stem-cell biotechnology, miRNAs

## Abstract

The clinical usage of induced pluripotent stem cell (iPSC)-derived regenerative medicine products is limited by the possibility of residual undifferentiated cells forming tumours after transplantation. Most of the existing quality control tests involve crushing of cells. As a result, the cells to be transplanted cannot be directly tested, thereby increasing the cost of transplantation. Therefore, we tested a highly sensitive and non-disruptive quality-testing method that involves measuring microRNAs (miRNAs) in culture supernatants released by cells. By measuring miR-302b in the culture supernatant, residual iPSCs were detected with higher sensitivity than by measuring *LIN28* (*Lin-28* Homolog A) in the cells. To use this method, we also monitored the progression of differentiation. Our novel highly sensitive and non-disruptive method for detecting residual undifferentiated cells will contribute to reducing the manufacturing cost of iPSC-derived products and improving the safety of transplantation.

## Introduction

Extensive research on the usage of iPSCs is being conducted in the field of regenerative medicine. The major hurdle in using iPSC-derived regenerative medicine products clinically is the possibility of residual undifferentiated cells forming tumours after transplantation^1,2^. Numerous studies have been conducted to remove unexpected cells such as undifferentiated cells from iPSC-derived target differentiated cells^3–6^. Most of the existing quality control tests for detecting undifferentiated cells in iPSC-derived products involve crushing of cells^3,4,7–9^; as a result, the cells to be transplanted cannot be tested directly, which in turn increases the cost of the procedure. Current commonly used methods to detect residual undifferentiated cells are as follows: detecting undifferentiated cell-specific protein using flow cytometry, measuring the levels of *Lin*-28 Homolog A (*LIN28A*), and colony formation assay (CFA)^2,7^; however, they require cells for testing.

MicroRNAs (miRNAs), non-coding RNAs with approximately 22 bases, are involved in cell fate decision and function^10–12^. miRNAs are released into body fluids such as blood and urine, and cell-type-specific miRNAs have been studied as biomarkers for pathological conditions such as cancer and diabetes^13,14^. The miR-302 family, miR-367, miR-371, miR-372, and miR-373 have been identified as miRNAs specific to embryonic stem cells (ESCs) and iPSCs^15–17^. Furthermore, miRNAs have been reported to be released into the culture supernatant by cells in a state bound to Ago2 or encapsulated in exosomes^18^. Currently, there is a need for a method to extract and measure miRNA with high efficiency for detecting miRNA from a few undifferentiated cells in iPSC-derived regenerative medicine products. Herein we report a method for non-disruptive detection of undifferentiated cells using miRNAs in culture supernatant as biomarkers.

## Results

### Extraction method for miRNA from culture supernatants

**Figure 1:**
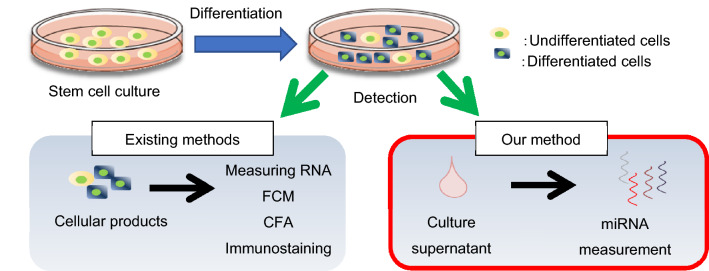
Schematic representation of the concept of our novel method for detecting residual undifferentiated cells using miRNAs in culture supernatants.

**Figure 2:**
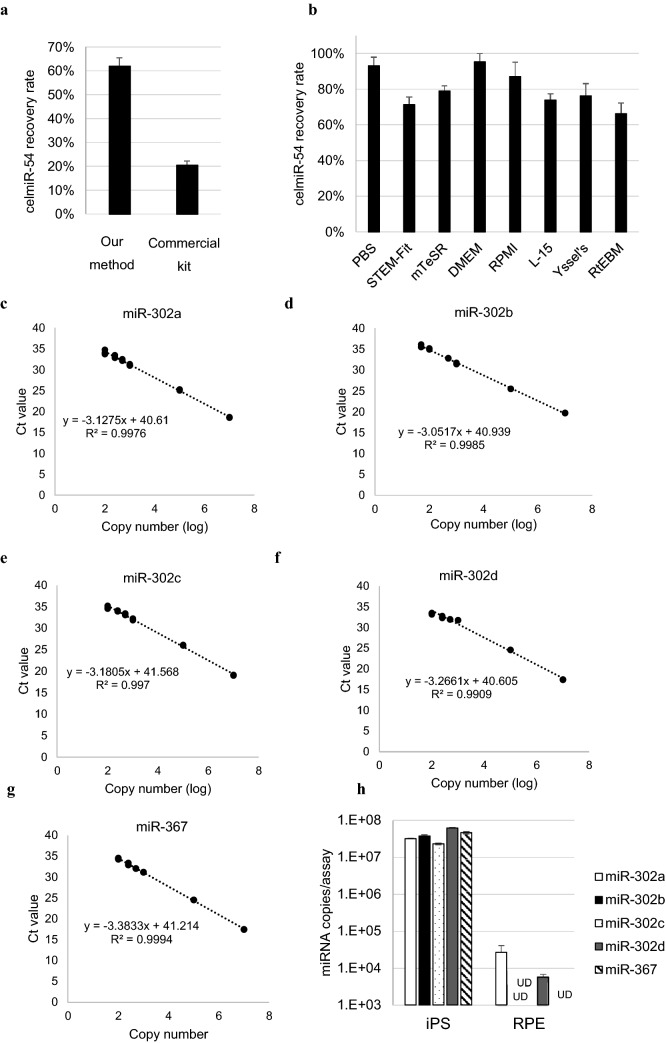
Highly efficient method to extract and detect miRNAs in culture supernatants was developed. (**a**) The recovery rate of spiked cel-miR-54 in PBS using our method and a commercially available kit, which is used to extract miRNAs using columns. (**b**) The recovery rate of spiked cel-miR-54 in various culture media using our method. (**c**–**g**) Calibration curves for each miRNA, plotted using the measured Ct values from 100 copies or 50 copies to 10^7^ copies, and the theoretical copy number (log). The slope and R^2^ value for each calibration curve are presented within the graph. (**h**) Level of each miRNA detected in the culture supernatant of iPSCs and RPE cells. *UD: no miRNA detected; error bar* =  + *3SD*.

We hypothesised that measuring miRNAs in the culture supernatant would enable the development of a highly sensitive and non-disruptive quality testing method (Fig. 1). First, we optimised an extraction method for miRNAs specifically from the culture supernatants. Using phosphate-buffered saline (PBS) spiked with a known amount of synthesised cel-miR-54 as a sample, a commercially available small RNA extraction column could be used to extract approximately 20% of spiked miRNA (Fig. 2a). On the contrary, we developed an miRNA extraction method specialised for culture supernatants that could recover approximately 60% of the spiked miRNA (Fig. 2a).

We confirmed the miRNA extraction efficiency in different types of media: STEM-Fit and mTeSR, which are generally used as an iPSC culture medium; Dulbecco’s modified Eagle medium (DMEM), Roswell Park Memorial Institute medium (RPMI), and Leibovits medium (L-15 medium), which are commonly used for other cell cultures; Yssel's serum-free T cell medium, which is a medium for T cells; and RtEBM, which is a medium for retinal pigment epithelium (RPE) cells. Each medium was spiked with synthetic cel-miR-54 to determine miRNA recovery rate. The results confirmed that our method extracted miRNA with a high recovery rate of more than approximately 70% from these seven media (Fig. 2b).

### miR-302b is an undifferentiated cell marker

The miR-302 family members and miR-367 (miR-302/367), which have been reported to be expressed abundantly in undifferentiated cells, such as iPSCs and ESCs^15^, were examined as potential markers for detecting undifferentiated cells. miR-371, miR-372, and miR-373 are also known as iPSC and ESC markers; however, their expression was lower than miR-302/367 expression (Supplementary Fig. 1). The sequences of miR-302/367 are shown in Table 1. We constructed quantitative reverse-transcription PCR (qRT-PCR) assay systems for these miRNAs and confirmed by following the MIQE guidelines^19^. The linear dynamic ranges were from 50 to 10^7^ copies for miR-302b and from 100 to 10^7^ copies for the other four miRNAs (Fig. 2c–g). The PCR efficiency ranged from 97.5 to 112.7%. These trials were performed three times for each miRNA, and we confirmed that the lowest concentrations were detected at 100%. By comparing the expression of miR-302/367 in culture supernatants of iPSCs and RPE cells, miR-302b, miR-302c, and miR-367 were detected specifically in the iPSC supernatant (Fig. 2h). miR-302b was not detected in RPE cells but was the most abundant in iPSCs. As miR-302b was the best marker for undifferentiated cells, we decided to carry out all further experiments using it.

**Table 1:** Sequences of the miR-302 family members and miR-367.

miR-302a	UAAGUGCUUCCAUGUUUUGGUGA
miR-302b	UAAGUGCUUCCAUGUUUUAGUAG
miR-302c	UAAGUGCUUCCAUGUUUCAGUGG
miR-302d	UAAGUGCUUCCAUGUUUGAGUGU
miR-367	AAUUGCACUUUAGCAAUGGUGA

**Figure 3:**
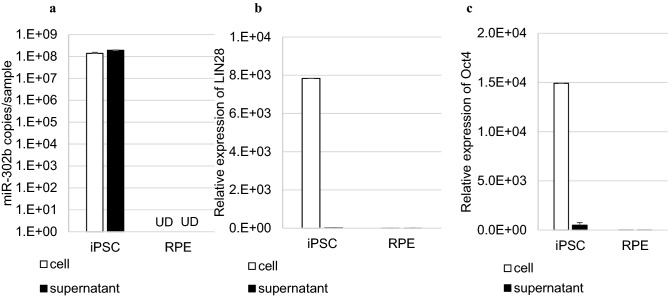
miRNA was abundantly secreted into the culture supernatant. The expression level of (**a**) miR-302b, (**b**) *LIN28*, and (**c**) *Oct4* in 10^5^ iPSCs and 10^5^ RPE cells and in 2 mL of their culture supernatants. For *LIN28* and *Oct4*, the vertical axis is the relative expression level as the expression level in RPE cells was 1. The level of miRNAs in the supernatant was corrected by multiplying the detected value with 20, because only 100 μL in 2 mL of the culture supernatants was used as a sample. As the cells were extracted from whole cells, such a correction was not performed. *UD: no miRNA detected; error bar* =  + *3SD*.

Next, we confirmed the extent of release of the undifferentiated cell-specific miRNAs in the culture supernatant. Using iPSCs and RPE cells, we compared the expression levels of *LIN28* and *Oct4* (also known as Pou5f1), which are well-known undifferentiated cell markers, and miR-302b in cells and the culture supernatant. We compared the amount of nucleic acids in 10^5^ iPSCs or RPE cells, and in 2 mL of their culture supernatants. Approximately 1.4 × 10^8^ copies of miR-302b were expressed in 10^5^ iPSCs (Fig. 3a). The *LIN28* and *Oct4* levels in iPSCs were approximately 8,000 times higher than those in RPE cells (Fig. 3b,c). In iPSCs, the *LIN28* and *Oct4* levels detected in the supernatant were 0.3% and 5.7% of intracellular expression, respectively. In contrast, approximately 6.0 × 10^7^ copies of miR-302b were released into the iPSC supernatant (Fig. 3a), which was half of the intracellular expression level. *LIN28* and *Oct4* were also detected at negligible levels in RPE cells and their culture supernatant, but miR-302b was not detected in them (Fig. 3a–c).

### Dynamics of miRNAs in the culture supernatant during culture and storage were confirmed

**Figure 4:**
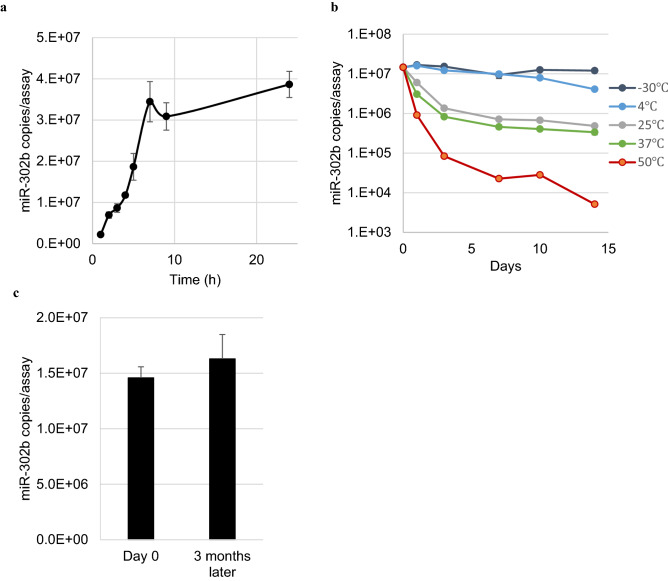
miRNAs in the culture supernatant were found to be stable. (**a**) Changes in the level of miR-302b in the culture supernatant observed up to 24 h after changing the medium. The graph shows data from three independent experiments. (**b**) The collected culture medium was stored at various temperatures for 2 weeks, and the change in the level of miR-302b was noted. Day 0 is the day of collection of the culture medium. (**c**) When the collected medium was stored at − 80 °C, the level of miR-302b detected before and that after 3 months of storage were comparable. Hence, − 80 °C was used in all further experiments. *Error bar* =  + *3SD.*

To use miRNAs in culture supernatants as targets for quality testing, properties such as stability during storage are important. We quantified the change in miRNA levels in the culture supernatant after changing the medium to determine the optimal timing for collecting the culture supernatant. The level of miR-302b in the culture supernatant reached saturation between 7 and 24 h after changing the medium (Fig. 4a). Next, to determine the storage conditions for the culture supernatant, the collected media were stored at various temperatures, and the changes in the level of miRNA in the medium were noted. At 25 °C or higher, the levels of miRNAs decreased to less than 10% of the original level within 3 days (Fig. 4b). At 4 °C, 70% of miRNAs were detected after 1 week and 30% after 2 weeks (Fig. 4c). At − 30 °C, the miRNA levels of more than 80% of the level on the day of collection could be detected after 2 weeks (Fig. 4b). Furthermore, at − 80 °C, miRNA in the medium was stably detected even after 3 months (Fig. 4c). Therefore, we decided to collect the culture media 24 h after the medium change and store them at − 80 °C.

### Level of miR-302b in the culture supernatant decreased with the induction of differentiation from iPSCs to neural progenitor cells (NPCs)

**Figure 5:**
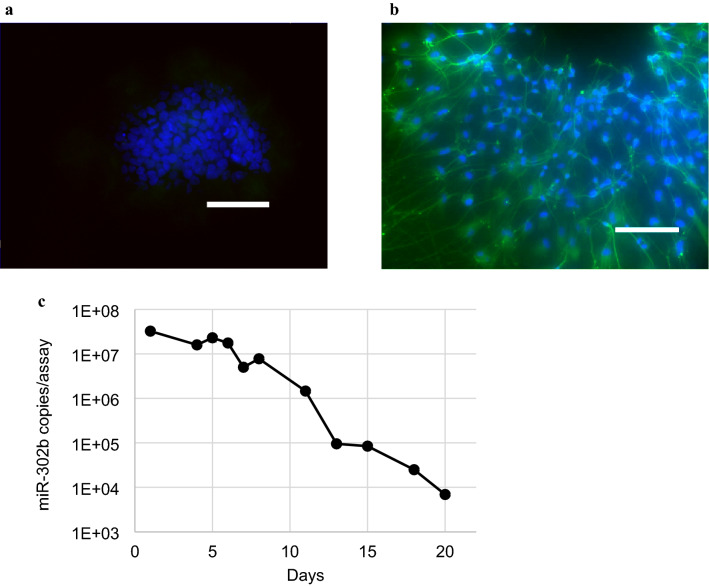
miR-302b expression level in the culture supernatant decreased as iPSCs were induced to differentiate to NPCs. The images of cells (**a**) before inducing differentiation, that is, iPSCs, and (**b**) on day 15 of the iPSC-derived NPC-induction process. Blue channel represents the nuclei and green is TUJ1. Scale bar is 50 μm. (**c**) The level of miR-302b in the culture supernatant continually decreased during the induction of iPSCs to NPC differentiation. *Error bar* =  ± *3SD*.

We examined whether the level of miR-302b in the culture supernatant reflects a change in the cell state. We checked the behaviour of miR-302b in the culture supernatant during the differentiation of iPSCs to NPCs. The neural marker TUJ1 (also known as TUBB3) was expressed on day 15 after the induction of differentiation, confirming that iPSCs were induced to differentiate into NPCs (Fig. 5a,b). During this process, miR-302b in the culture supernatant continually decreased, reaching 1/100 of the pre-differentiation level on day 15 and 1/1000 on day 20 (Fig. 5c).

### miR-302b measurement in the culture supernatant detected undifferentiated cells with a higher sensitivity than *LIN28* measurement

**Figure 6:**
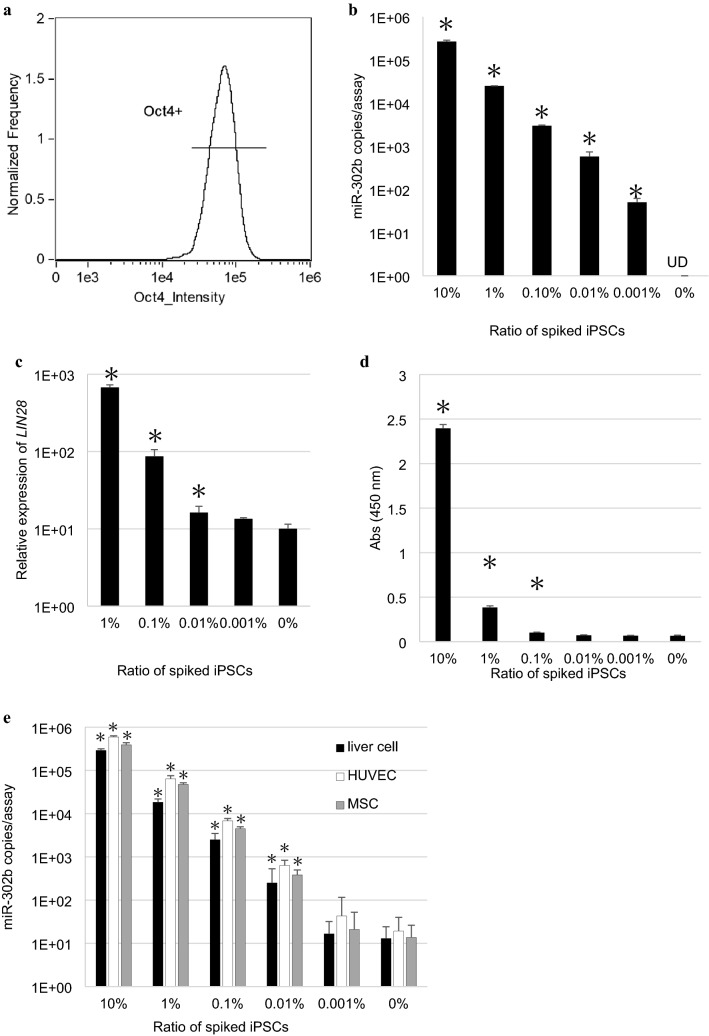
Sensitivity of detecting undifferentiated cells by measuring miR-302b in culture supernatant was 0.001%. (**a**) Oct4-positive rate of iPSCs used to develop the residual undifferentiated cell model was found to be 98.2%. (**b**) The detection sensitivity of miR-302b in the culture supernatant, (**c**) *LIN28* in the cells, and (**d**) Fuca1-2Galβ1-3GaINAc in the culture supernatant was compared in the same residual undifferentiated cell model in which 10^6^ RPE cells were spiked with the appropriate number of iPSCs. (**e**) Detection sensitivity of undifferentiated cells measured by detecting miR-302b in the culture supernatant of liver cells, HUVECs, and MSCs. *Error bar* =  + *3SD*. UD: no miRNA detected. *p value < *0.01, compared with 0%, Student’s t-test.*

To quantify the efficiency of detecting undifferentiated cells by miR-302b in culture supernatants, we established a residual undifferentiated cell model in which 10^6^ RPE cells were spiked with an arbitrary amount of iPSCs. To determine the accuracy of the model, we constructed a model using only iPSCs that maintain an undifferentiated state. Because iPSCs are prone to differentiate at the colony margin during culture^20^, it is possible that undifferentiated iPSCs are contaminated with iPSCs that have lost their undifferentiated state. Therefore, we confirmed the expression of Oct4 in each cell using an imaging flow cytometer, and found that the proportion of Oct4-positive cells was 98.2% (Fig. 6a). We then constructed our accurate residual undifferentiated cell model by measuring miR-302b in the supernatant, 0.001%, that is, 10 iPSCs could be detected in the RPE cell background (Fig. 6b). In contrast, the measurement of *LIN28* in the cells could only detect iPSCs up to 0.01% of RPE cells (Fig. 6c). A non-disruptive undifferentiated cell detection method for detecting H-type3 (Fuca1-2Galβ1-3GaINAc), a mucin-like o-glycan on the surface of iPSCs, with rBC2LCN lectin, has been reported^21^. We further measured H-type3 in the culture supernatant using the same samples, and achieved a performance of 0.1% (Fig. 6d). Furthermore, we checked the detection sensitivity of miR-302b in the supernatant in Clonetics™ human hepatocyte cell system (liver cell), human umbilical vein endothelial cell (HUVEC), and mesenchymal stem cell (MSC) backgrounds using mixed supernatants. Specifically, the culture supernatants of iPSCs and differentiated cells were mixed at an arbitrary ratio according to the number of each cells. In these mixed supernatants, the miR-302b level was below the detection limit in 0% and 0.001% iPSC samples, and the detection performance was 0.01% in all three backgrounds (Fig. 6e).

**Figure 7:**
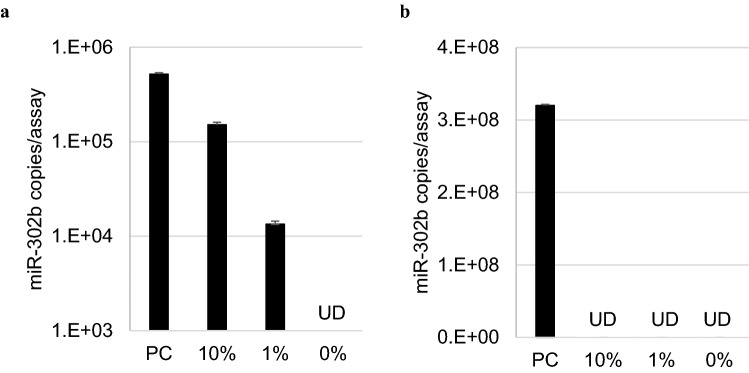
Measuring miR-302b in the culture supernatant allows the detection of iPSCs in a large number of cells. (**a**) 10^7^ HCT116 cells were spiked with iPSCs, and miR-302b was measured in the culture supernatant. (**b**) 10^7^ HCT116 cells were spiked with iPSCs and miR-302b was measured in the cells. *PC: positive control, which is the culture condition of only 10*^*6*^* iPS cells. UD: no miRNA detected. Error bar* =  + *3SD.*

A large number of cells is required for the transplantation of heart or liver cells generated from iPSCs, which are currently under clinical research. Nucleic acid extraction and PCR are inhibited when a target gene is detected in many cells. Therefore, we investigated the possibility of detecting miRNAs in the culture supernatant under scaled-up conditions. HCT116 cells (10^7^) spiked with 1% (10^5^ cells) or 10% (10^6^ cells) of iPSCs were seeded in a 10-cm dish, and miR-302b was extracted and measured from the cells and supernatant, respectively. All detached cells and 100 μL of the supernatant from 10 mL of the medium were used as samples. The positive control sample (PC) consisted of 10^6^ iPSCs, and miR-302b was detected in both cells and supernatant (Fig. 7). In the sample consisting of 0% iPSC sample, that is, only 10^7^ HCT116 cells, miR-302b was not detected in the cells or supernatant (Fig. 7). However, in the samples with HCT116 cells spiked with iPSCs, miR-302b was detected in the culture supernatant but not in the cells (Fig. 7).

## Discussion

In order to use iPSC-derived products, it is important to evaluate the tumorigenic residual undifferentiated cells, and various studies have been conducted with the aim to increase detection sensitivity. Most of the existing methods need the cell products to be crushed, even though it is desirable to inspect the cells to be transplanted. It is known that mutations occur in some of the iPSCs during culture^22^ and that the quality of iPSCs may change depending on the culturing skills, even if identical protocols are followed^23,24^. Because miRNAs are potential biomarkers released from cells, we hypothesised that cells could be evaluated non-disruptively by measuring the miRNAs in culture supernatants (Fig. 1). To improve the recovery rate of miRNAs in culture supernatants, with less impurities than in cells or blood, it was necessary to develop an miRNA extraction method specifically adapted to culture supernatants (Fig. 2a). As it takes a long time and various types of media to induce the differentiation of iPSCs to target cells, a method that can extract miRNAs with high recovery rates from various media is indispensable. Although our method can be used to extract miRNAs from various media with high recovery rates, the rates were variable (Fig. 2b). This was because the rates were compared based on the number of miRNA copies. When expressed in terms of threshold cycle (Ct) value, the CV was less than 1.5%, which indicates that our method is highly stable. In addition, miRNAs in the supernatant could be stably stored for a long period in a frozen state (Fig. 4c). This enabled us to compare the number of miRNAs in the supernatant of the initial cells with that after long-term induction of differentiation, and it is possible to outsource the quality control test instead of performing it only in the cell processing centre (CPC).

miR-302b was detected in both iPSCs and the culture supernatant, but not in RPE or HCT116 cells (Figs. 3a, 7). In addition, during the process of differentiation of iPSCs to NPCs, the miR-302b level decreased with the induction of differentiation (Fig. 5c). Therefore, miR-302b in the supernatant can be used as a specific marker for iPSCs. Our method to detect residual undifferentiated cells non-disruptively can be applied to not only the final quality control tests of the transplanted cells but also monitor changes in the nature of cells by measuring target cell-specific miRNAs during the differentiation and reprogramming processes. *LIN28* and *Oct4* are well known iPSC/ESC makers^2^. Their expression levels in iPSCs were approximately 8,000 times higher than that in RPE cells, and the *LIN28* and *Oct4* levels detected in the supernatant were 0.3% and 5.7% of the intracellular level, respectively (Fig. 3b,c). It has been reported that the mRNA encoding the nuclear protein cannot be brought to the extracellular space by exosomes^25^. These mRNAs in the iPSC supernatant were considered to have been from dead cells in the supernatants. In order to establish a simple miRNA recovery method, the step of centrifuging the culture supernatant before miRNA extraction to remove dead cells was not performed in this study. It has been confirmed that there was no significant difference in the level of miRNA extracted depending on the presence or absence of this step.

It is considered that *LIN28* is more sensitive for detecting undifferentiated iPSCs^7^. By measuring miR-302b in the supernatant, undifferentiated cells were detected with equal or higher sensitivity than by using the currently widely used *LIN28* measurement test. In a study in hepatic endoderm (HE) cells, which express *LIN28*, embryonic stem cell related (ESRG), chondromodulin (CNMD), and secreted frizzled related protein 2 (SFRP2) were better markers of residual undifferentiated cells from various differentiated cells^8^. By measuring these mRNAs using qPCR, undifferentiated cells have been detected with a performance of 0.005%, 0.025%, and 0.025%, respectively^8^. miR-302b has also been used to detect undifferentiated cells from various differentiated cells including liver cell line with a performance of 0.01–0.001%, respectively (Figs. 3, 5, 6, 7). This miRNA is highly and specifically expressed in human iPSCs^15^, and it has been used as a target for the elimination of residual undifferentiated cells^4^. Additionally, the expression of exogenous miR-302 cluster has an important role in reprogramming the cells to differentiate into iPSCs^26^. On the basis of these findings, miR-302b is a valuable marker for undifferentiated cells.

A quality control test method, called the highly efficient culture (HEC) assay, with improved CFA has been newly developed^27^. This assay can detect colonies formed from iPSCs using a highly efficient culture system, which favours the growth of iPSCs, and the limit of detection (LOD) has been reported to be 0.001%^27^. In addition, this assay has been improved with a sorting system, and the LOD was reportedly 0.0002%^9^. The disadvantages of this assay are it requires 1 week and a large amount of samples used for test. To achieve the same sensitivity as this improved assay, our method is required to be five times more sensitive. Further improvement in sensitivity of miRNA detection methods using nucleic acids, with the classical method using precipitation with ethanol or polyethylene glycol (PEG), is relatively easy.

A non-disruptive method that measures glycans has been reported, with a theoretical sensitivity of 0.005% (500 iPSCs/10^7^ differentiated cells/10 mL)^14^. We compared the performance of our miRNA detection method and the glycan-based method in detecting undifferentiated cells using the same samples. We found that the sensitivity of our miRNA detection method was higher than that of the glycan-based method (Fig. 6b,d).

A method to measure mouse-specific miRNAs (miR-292 and miR-294) in the cell supernatants for detecting mouse and human iPS/ES cells has been reported^28^. For this method, more than 5000 human iPSCs are needed to detect these miRNAs^28^. Our method could detect 10 iPSCs and the performance of detection is considerably higher than that of this method. This high sensitivity was achieved because we selected miR-302b, which is particularly abundant in the supernatant among the miRNAs reported as human iPS/ES cell markers, and developed a method for extracting miRNA from the culture supernatant with high efficiency (Supplementary Fig. 1, Fig. 1A)^15–17^.

The transplantation of iPSC-derived products requires a large number of cells. However, as the number of cells increases, the concentration of nucleic acids in the sample usually increases, which in turn increases the inhibition of nucleic acid extraction and reverse transcription reactions. Therefore, it is difficult to extract and detect target nucleic acid molecules from high-concentration nucleic acids in large amounts of cells. As our miRNA detection method uses the extracted samples that does not contain many long nucleic acids, it is possible to detect undifferentiated cells using miRNAs even in a large-scale culture system (Fig. 7). Therefore, it is unlikely that small-scale samples for testing would be required, and quality testing can be performed directly using culture supernatants from cells used for transplantation. Owing to improved treatment outcomes^29^, iPSC-derived products are increasingly being formed into structures, such as sheets^30^, spheroids^31^, and organoids^32^. Our method is also applicable to spheroids (Supplemental Fig. 2); therefore, we will further investigate whether it is effective for other structures such as organoids.

In summary, our method detected 0.001% of iPSCs in differentiated cells using undifferentiated cell-specific miRNA in the culture supernatant. It is a novel highly sensitive and non-disruptive undifferentiated cell detection method. This method should contribute to cost reduction in the manufacture of iPSC-derived products and improve the safety of transplantation.

## Materials and methods

### Cell culture

Human iPSC cell line (201B7) was purchased from the Centre for iPS Cell Research and Application (CiRA) and maintained following CiRA’s protocol^33^, on iMatrix511 (892011, NIPPI) in StemFit (AK02N, Ajinomoto) at 37 °C in a humidified incubator with 5% CO_2_. Human primary retinal pigmental epithelial (RPE) cell line was purchased from Lonza and maintained as described in Lonza’s catalogue^34^ in RtEGM with RtEGM SingleQuots (Lonza) at 37 °C in a humidified incubator with 5% CO_2_. The liver cell line (h NHEPS™ Adherent Cells, CC-2591) was purchased form Lonza and maintained following the instructions of Lonza^35^ in HBM™ (hepatocyte basal medium) or HMM™ (hepatocyte maintenance medium) at 37 °C in a humidified incubator with 5% CO_2_. Human umbilical vein endothelial cell (HUVEC, C2517A) line was purchased from Lonza and maintained using the VascuLife VEGF Medium Complete Kit (Kurabo) at 37 °C in a humidified incubator with 5% CO_2_. Mesenchymal stem cell (MSC) line (hMSC-Human Mesenchymal Stam Cells, PT-2501) was purchased from Lonza and maintained using the MECGM-CD BulletKit (Lonza) at 37 °C in a humidified incubator with 5% CO_2_. All cells were cultured in six-well plates with 2 mL of medium per well.

HCT116 cell line was purchased from RIKEN BioResource Research Center and maintained in low-glucose DMEM with 10% (v/v) foetal bovine serum (FBS) at 37 °C in a humidified incubator with 5% CO_2_. HCT116 cells were cultured in 10-cm dishes with 10 mL of medium.

Supernatants from the cells were collected 24 h after changing the media.

### Induction of NPC differentiation

The STEMdiff™ SMADi Neural Induction Kit (ST-08581, STEMCELL) was used to induce the differentiation of iPSCs into NPCs according to the STEMCELL manual^36^. iPSCs were cultured according to the instructions in the manual^36^, in the mTeSR (ST-85850, STEMCELL) medium. To confirm differentiation into NPCs, the cells were fixed using 4% paraformaldehyde (PFA) and stained with TUJ1 antibody (801213, BioLegend) and DAPI.

### Preparation of residual undifferentiated cell model

iPSCs (7 × 10^4^) were fixed with 4% PFA and stained with the PE anti-Oct4 (Oct3) antibody (653704, BioLegend). The number of Oct4-positive cells was determined using Amnis™ ImageStream™ X MkII (Luminex). PE Mouse IgG2b (400314, BioLegend) was used to set the gate. Hoechst stain was used to stain the nucleus.

After seeding iPSCs (described above)^33^, the differentiated cells were seeded and cultured in StemFit AK03 at 37 °C in a humidified incubator with 5% CO_2_. Twenty-four hours later, the media were collected and stored at − 80 °C. The cells were then washed twice with PBS and treated with Accutase (12679-54, Nakarai). The detached cells were centrifuged, the supernatant was removed, washed with PBS, and stored at − 80 °C as cell samples.

### RNA extraction and quantitative RT-PCR

The total RNA was isolated from whole cells or 100 μL of culture supernatant using a High Pure RNA Isolation Kit (11828665001, Roche Diagnostics). One-step RT-PCR, using 1/50 of the extracted RNA using the TaqMan Fast Virus 1-Step Master Mix (4444436, Thermo Fisher Scientific), was performed on a 7500 Fast real-time PCR System (Applied Biosystems). The reaction mixtures were incubated in a 96-well plate at 50 °C for 20 min, 95 °C for 5 min, followed by 40 cycles at 95 °C for 15 s and 60 °C for 1 min. The Ct value was defined as the fractional cycle number at which fluorescence passed the fixed threshold. The PCR primers and probes were purchased from Thermo Fisher Scientific.

### miRNA extraction and quantitative RT-PCR

miRNAs were isolated from whole cells or 100 μL of culture supernatant using the modified boom method^37^. The cells or culture supernatants were solubilised in 312 μL of buffer (40% guanidine thiocyanate, 2.5% dithiothreitol, 50 mM Tris–HCl (pH 8.0), 20% Tween20, and nuclease free water). Thereafter, 1 mL of tetraethylene glycol dimethlyl ether and 75 μL of magnetic beads (Sicastar-M, 39–00-153, CoreFront) were added to the samples, and nucleic acids in the samples were absorbed by the beads. After washing with 80% ethanol buffer (80% ethanol, 20 mM NaCl, 2 mM Tris–HCl (pH 7.5), and nuclease free water) twice, nucleic acids were eluted in 100 μL of nuclease free water. The High Pure miRNA Isolation Kit (05080576001, Roche Diagnostics GmbH) was used as a commercial miRNA extraction kit. Next, 16 μL of the purified 100 μL miRNA was reverse-transcribed into cDNA using MultiScribe reverse transcriptase (4311235 Applied Biosystems). The reaction mixtures were incubated in the GeneAmp PCR System 9700 (Applied Biosystems) in a 96-well plate for 30 min at 16 °C, 30 min at 42 °C, 5 min at 85 °C, and then held at 4 °C. Real-time PCR was performed using the Hot Start ExTaq PCR enzyme (RR006B, TaKaRa) following a standard TaqMan PCR protocol on a 7500 Fast Real-Time PCR System (Applied Biosystems). The reaction mixtures were incubated in a 96-well plate at 95 °C for 10 min, followed by 40 cycles at 95 °C for 15 s and 60 °C for 1 min. The Ct value was defined as the fractional cycle number at which fluorescence passed the fixed threshold. The Ct values were converted into absolute copy numbers using a standard curve of synthetic miRNAs. Stem-loop RT primers, PCR primers, and probes were designed as previously described^38^. For the analysis, the level of miRNAs in the supernatant was corrected by multiplying the detected value with 20, because only 100 μL in 2 mL of the culture supernatants was used as a sample.

### Measurement of H-type3

H-type3 was detected using ELISA in 50 μL of the culture supernatant with the Human ES/iPS Cell Monitoring Kit (299-78301, Wako), according to the manufacturer’s instructions. The absorbance of the samples was measured at 450 nm using Infinit F200 Pro (Tecan).

### Statistical analysis

All real-time PCR experiments were performed in triplicate, and all experiments were repeated several times. Data are presented as mean ± 3 SD of three or more independent experiments. Student’s *t*-test was used for the statistical analyses of the experimental results.

## Supplementary Information

Supplementary Figures.

## Data Availability

The datasets generated during and/or analysed during the current study are available from the corresponding author on reasonable request.
