# Supplementary material for: Highly sensitive and non-disruptive detection of residual undifferentiated cells by measuring miRNAs in culture supernatant
Source: Sci Rep. 2022 Jun 20;12:10351. doi: 10.1038/s41598-022-14273-z (PMC9209417; doi:10.1038/s41598-022-14273-z)
Supplement: Supplementary file 1 — Supplementary Figures. [file 41598_2022_14273_MOESM1_ESM.docx]

**Highly sensitive and non-disruptive detection of residual undifferentiated cells by measuring miRNAs in culture supernatant**

**Kanako Masumoto, Yuki Aihara^*^, Mao Miyagawa Kuroishi, Natsuki Maeda, Yumiko Sakai, Yuma Oka, Yusuke Takahashi, Kenta Oda, and Masatoshi Yanagida**

Sysmex Corporation, Central Research Laboratories, 4-4-4 Takatsukadai, Nishi-ku, Kobe, 651-2271, Japan

**Supplemental Figure 1**. Expression levels of miR-302/367, miR-371, miR-372, and miR-373 in the culture supernatant of iPSCs. iPSCs (10^5^) were seeded in 6-well plates coated with iMatrix511 and maintained with 1.5 mL of StemFit. After 24 h, the supernatants were collected and centrifuged at 1,500 *g* for 10 min. The miRNAs were extracted from 50 μL of the supernatants using the High Pure miRNA Isolation Kit (05080576001, Roche Diagnostics GmbH). One microliter of the purified miRNA was reverse transcribed into cDNA using the TaqMan MicroRNA Reverse Transcription Kit (4366597, Thermo Fisher Scientific). The reaction mixtures were incubated in a 96-well plate in GeneAmp PCR System 9700 (Applied Biosystems) for 30 min at 16°C, 30 min at 42°C, 5 min at 85°C, and then held at 4°C. Real-time PCR was performed using TaqMan Universal Master Mix II with UNG (4440042, Thermo Fisher Scientific) on a 7500 Fast Real-Time PCR System (Applied Biosystems). The reaction mixtures were incubated in a 96-well plate at 95°C for 10 min, followed by 40 cycles at 95°C for 15 s and 60°C for 1 min. The threshold cycle (Ct) value was defined as the fractional cycle number at which fluorescence passed the fixed threshold. Each set of primers and probes (the TaqMan MicroRNA Assay) for each miRNA was purchased from Thermo Fisher Scientific. The vertical axis is the relative expression level of each miRNA when Ct value 40 is expression level 1. *UD: no miRNA detected. Error bar = +3SD*.

**Supplemental Figure 2**. miR-302b was also detectable in the culture supernatant from 3D cultures. The amount of miR-302b in the supernatant when 1.5×10^6^ iPSCs were cultured in a normal planar culture (6 well plate) or 3D culture using a microwell (6 well plate). All methods are the same as those described in the manuscript. *Error bar = +3SD*.
